# Medication adherence support of an in-home electronic medication dispensing system for individuals living with chronic conditions: a pilot randomized controlled trial

**DOI:** 10.1186/s12877-020-01979-w

**Published:** 2021-01-14

**Authors:** Mubashir Aslam Arain, Armghan Ahmad, Venus Chiu, Lorena Kembel

**Affiliations:** 1 Health Systems Evaluation and Evidence, Alberta Health Services, 10301 Southport Lane SW, Calgary, Alberta Canada; 2 Department of Family Medicine, University of Alberta, Edmonton, Alberta Canada

**Keywords:** Medication dispenser, Medication adherence, Health conditions, Aging and older adults, Randomized controlled trial

## Abstract

**Background:**

Medication adherence is challenging for older adults due to factors such as the number of medications, dosing schedule, and the duration of drug therapy. The objective of this study was to examine the effectiveness of an in-home electronic medication dispensing system (MDS) on improving medication adherence and health perception in older adults with chronic conditions.

**Methods:**

A pilot Randomized Controlled Trial (RCT) was conducted using a two-arm parallel assignment model. The intervention group used an MDS as their medication management method. The control group continued to use their current methods of medication management. Block randomization was used to assign participants into the intervention or control group. The inclusion criteria included 1) English speaking 2) age 50 and over 3) diagnosed with one or more chronic condition(s) 4) currently taking five or more oral medications 5) City of Calgary resident. Participants were recruited from a primary care clinic in Alberta, Canada. The study was open-label where knowledge about group assigned to participants after randomization was not withheld. Medication adherence was captured over a continuous, six-month period and analyzed using Intention-to-Treat (ITT) analysis.

**Results:**

A total of 91 participants were assessed for eligibility and 50 were randomized into the two groups. The number of participants analyzed for ITT was 23 and 25 in the intervention and control group, respectively. Most of the demographic characteristics were comparable in the two groups except the mean age of the intervention group, which was higher compared to the control group (63.96 ± 7.86 versus 59.52 ± 5.93, *p*-value = 0.03). The average recorded adherence over 26 weeks was significantly higher in the intervention group than the control group (98.35% ± 2.15% versus 91.17% ± 9.76%, *p* < 0.01). The self-rated medication adherence in the intervention group also showed a significant increase from baseline to 6-month (Z=-2.65, *p* < 0.01). The control group showed a non-significant increase (Z=-1.79, *p* = 0.07).

**Conclusion:**

The MDS can be an effective, long-term solution to medication non-adherence in older adults experiencing chronic conditions and taking multiple medications. The technology induces better consistency and improvement in medication taking behaviour than simple, non-technological intervention.

**Trial registration:**

Registered with ClinicalTrials.gov on April 09, 2020 with identifier NCT04339296.

**Supplementary Information:**

The online version contains supplementary material available at 10.1186/s12877-020-01979-w.

## Background

It is unequivocal that the world population is on the trajectory of rapid aging. It is estimated that by 2050, one in six people in the world will be 65 years old or older [1]. As life expectancy improves globally, so does the prevalence of chronic morbidity in older people. The prescription of multiple medications and complex medication regimens are common among older adults to treat and prevent complications associated with chronic diseases. In Canada and the United States, 31 and 35% of older adults aged 60–79 use five or more prescription drugs, respectively [2]. Canadian older adults aged 65 and older and living in the community, on average, are prescribed 6.7 medications [3]. As a result, medication adherence is particularly challenging for older adults due to medication regimen factors, such as the number of medications, dosing schedule, and the duration of drug therapy [4].

Adherence to drug therapy is defined as the extent to which an individual is able to take medications as recommended by health care providers [5]. The full benefit of pharmacotherapy can only be realized when patients take medications as prescribed. Non-adherence, conversely, poses a challenge for both the patients and the healthcare system. Patients experience more disease-related complications, poorer health outcomes, lower quality of life, and elevated risk of death [6]. The annual economic impact of non-adherence on the healthcare system is substantial, costing $7–9 billion in Canada [7], and over $100–300 billion in the United States [6, 8]. Despite profuse research on the negative consequences of medication non-adherence, a systematic review reported that up to 55% of community-dwelling older adults (65 and above) taking multiple medications for their chronic diseases is non-adherent [9].

Strategies to improve medication-taking behaviour in community-dwelling older adults abound, such as providing reminders (e.g., alarms), modifying medication packaging (e.g., blister packs, pill boxes), and following-up with individuals (e.g., home visits) [10]. Advances in technology have allowed for less resource-intensive dissemination and delivery of alternative interventions. For example, telephone calls, text messaging, internet-based programs, telehealth devices, and pharmaceutical databases have been increasingly utilized to educate, monitor, and remind patients [11, 12]. Telehealth devices offer a tremendous opportunity for home health monitoring of medication adherence and vital sign measurements [13]. Telehealth devices are promising solutions for the aging population to manage chronic conditions safely and conveniently but acceptability of utilization vary among users [13, 14]. Similarly, medication dispensing technology is a comparatively novel innovation that integrates multiple forms of intervention into one device. Its core features include dispensing pre-organized medications and providing reminders. More complex designs offer the ability to monitor, promote, or intervene with users’ medication administration behaviour.

Research on mediation dispensing technology and its use among community-dwelling older adults has only commenced in recent years. Despite the high costs and mixed preferences and needs among study participants, user experience is generally positive [15–17]. Older adults find the devices acceptable [15, 16, 18], easy to use [15, 16, 18, 19], and supportive of individuals’ daily activities [15, 16]. Users further report a perceived improvement of medication adherence [15, 16, 19] and health outcomes [15]. Subjective experiences aside, limited quantitative studies have been conducted to justify the perceived effectiveness of the technology in improving mediation adherence and health in the older population. Moreover, even fewer studies have been conducted in controlled settings to rigorously determine the cause-effect relationship between the intervention and outcomes [20]. In response to calls for randomized controlled trials (RCTs) to quantitatively evaluate the validity of previous findings [15, 16, 19], this pilot study aims to examine the effectiveness of an in-home electronic medication dispensing system (MDS) on improving medication adherence and health perception in community-dwelling older adults with chronic conditions.

## Methods

### Design

The investigators conducted a pilot RCT using a two-arm parallel assignment model with 1:1 allocation. The intervention group used an MDS as their medication management method. The control group continued to use their current methods of medication management, such as blister packs, pill organizers, and plastic prescription vials. This study was conducted in Calgary, Alberta, Canada. The data for each participant was collected over a continuous, six-month period between July 2019 and May 2020.

The study was a proof of concept for which no formal sample size calculation was required [21]. The target sample size was 100 study participants with 50 participants each randomly assigned to either the intervention or the control group. Block randomization was used to assign participants into the intervention or control group. Block randomization is the recommended method for sample sizes of 50 or less in each group [22]. This randomization method was utilized to balance the groups in terms of the number of subjects they contain and the distribution of potential confounding variables. The study was open-label where knowledge about group assigned to participants after randomization was not withheld from parties involved in the study. The blocks varied in sizes (10, 12, and 14) to reduce the predictability of the allocation assignment and to keep the participants and investigators blind to the size of each block [22]. A project assistant (not involved in data collection or analysis activities) produced computer-generated sets of random allocations using a website in advance of the study [23]. The randomization sequence was concealed in consecutively numbered opaque envelopes. Once the participant consented to be included in the study, each participant was irreversibly randomized into a group by a research coordinator through opening the next concealed envelope containing their assignment.

The cost of using the  MDS was covered through the funding program during the study. In addition, participants received a $50 gift card for participating in the study. Ethics approval was granted by the Human Research Ethics Board at the University of Alberta with identification PRO 00087782. The trial is also registered with ClinicalTrials.gov with identifier NCT04339296 and adheres to Consolidated Standards of Reporting Trials (CONSORT) guidelines [24].

### Description of intervention

The MDS is a non-surgical device approved by Health Canada under Class I of Medical Device Establishment License. The MDS aimed to optimize medication adherence by dispensing pre-packaged medications on time with reminder alerts while tracking medications and changes to symptoms experienced by the users. Medications were organized into pouches by day and time of administration, and labelled with participant's name, medication name and dosage. The sound of the reminder alerts amplified if the medication pouches weren’t dispensed from the system by the user. Moreover, the MDS provided flexibility to users by allowing early dispensing of medications, if required. The pharmacy was able to remotely monitor medication adherence through an electronic monitoring system called AdhereNet. AdhereNet is a data coordination platform linking pharmacists, patients and care teams to help users manage drug complexity and medication administration in real-time [25]. The pharmacy also had the ability to monitor the well-being of users through setting up personalized and condition-specific questions. The caregivers were electronically connected and had access to user’s data using the caregiver mobile application. The MDS also has the capability to connect Bluetooth embedded devices to measure and monitor daily vital signs such as glucometer, blood pressure monitor, and weight scale; but, this was beyond the aim of the pilot study.

The task of using the MDS required individuals to have cognitive skills for daily decision making. There was a risk that the MDS might not function properly, or that participants might not clearly understand the device functionalities. Also, the participants might not comprehend the steps or actions required to fully administer their medication correctly. In order to address the perceived risks and mitigate harm, study participants that were using the MDS received a one-hour training by pharmacy staff. Additionally, to ensure the proper functioning of the MDS, participants were provided with 24- h technical support for issues that could be self-resolved through telephone support. In other circumstances such as the MDS fails to function, participants had access to their medications by taking out the medication cartilage box which was labeled with medication name and timing.

### Eligibility criteria

The eligibility criteria for participant recruitment included 1) English speaking 2) age 50 and over 3) diagnosed with one or more chronic condition(s) 4) currently taking five or more oral medications 5) City of Calgary resident [15]. Firstly, participants were required to be English speaking so they understood the study, their role and were able to effectively communicate with the research team to generate the evidence required for the study. Secondly, participants were required to be age 50 and over to have a representative sample of the aging population. In addition, participants needed to be diagnosed with at least one chronic condition and taking a minimum of five oral medications because the MDS acted as a medication reminder and management support system for individuals on multiple medication regimens. Moreover, only individuals from the City of Calgary were eligible because only one pharmacy adopted the technology and could only manage participants within the city limits. The exclusion criteria restricted individuals with moderate to severe cognitive impairment from participating in the study [15]. These individuals would lack sufficient cognition and decision making ability to provide informed consent and operate the MDS safely [15].

### Participant recruitment

Participants were recruited from a Primary Care Clinic in Calgary, Alberta during the period of June to October 2019. Prior to study recruitment, the clinic compiled a list of potential study participants from their patient panel based on two filters of age and medication criteria. Each healthcare provider screened the list of their patients for cognitive function and study suitability and concluded 643 were a good fit.

During the recruitment period, two strategies were utilized to inform potential eligible participants about the study. First, the clinic mailed a total of 517 invitation letters to potentially eligible participants on behalf of the research team to increase awareness and participation. The interested participants were required to directly contact the research coordinator for study enrollment. Second, potentially eligible participants with scheduled appointments at the clinic were flagged by the clinic administrative staff so the healthcare provider could inform them about the study. Interested participants were directed to the research coordinator on-site for study enrollment. A written consent was obtained from all participants enrolled in the study.

**Fig. 1:**
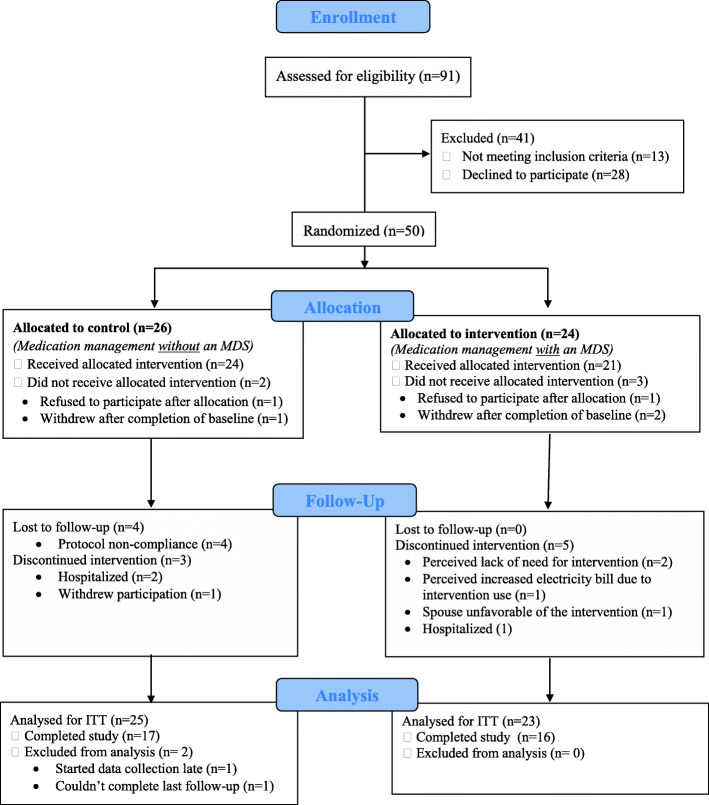
CONSORT flow diagram of participants in the pilot RCT

For the duration of the study, the intervention group participants were asked to change their current pharmacy to a new pharmacy that was partnering with the MDS supplier. The control group participants were not required to change their pharmacy. Out of the 91 participants assessed for eligibility (Fig. 1), 28 of the eligible patients refused to participate for the following reasons: 1) time constraints for study commitment 2) fear of using technology 3) no previous issue(s) or challenge(s) with managing their medications 4) preferred not to change pharmacy 5) The post-study fee of using the MDS was a barrier to participating in the study.

### Data collection

Participants’ demographics, health history and medication management information were obtained through baseline assessment once they consented to participate in the study and have been randomized into a group. The baseline questionnaires were often completed at the clinic, over the telephone or through home visits. A monthly follow-up was conducted for six months over the phone for all participants to record changes in self-reported medication management and health status in the past month. Both baseline and follow-up data were collected and managed using Research Electronic Data Capture (REDCap) tools hosted at Alberta Health Services [26, 27].

The intervention group adherence recording was obtained from AdhereNet. The MDS adherence was defined as the percentage of medications dispensed from the system at the prescribed time. The user had a two-hour window to remove the medication pouch. If dispensed, they are given a score of 100 for the particular medication time. If not removed, the user was considered non-adherent and given a score of 0. The medication adherence was based on an assumption that medication was administered by the user once the pouch was removed from the MDS. Weekly medication adherence was calculated by taking the seven day average of daily dispensing adherence values (see Additional file 1 for equation and calculation).

The control group adherence was measured through monthly medication calendars recorded by participants. Each participant received customized calendars based on their medications timings. Participants were required to mark “X” on the calendar on the day and time they missed taking their medication. Each control participant was required to complete and mail back the calendars through pre-paid postage envelopes on a monthly basis as part of the study protocol. Reminders for control group participants to send back their medication calendar for the previous month was done during follow-up phone calls. If there were any changes in medication timings, changes were recorded during follow-up and revised calendars were mailed-out again. The control group calendar data was entered into an Excel spreadsheet with the predetermined equation for adherence calculation. Weekly medication adherence was calculated using the same approach as the intervention group.

Multi-measure approach to measuring medication adherence is the recommended practice to increase the accuracy of adherence measurements [28]. As such, in addition to the recorded adherence, a second medication adherence measure was employed. The investigators developed a self-reported medication management and adherence, and health status questionnaire. The self-reported questionnaire was completed in both groups at baseline and follow-ups for comparison purposes. The adherence question asked participants to rate their medication adherence on a scale of 1 (non-adherent) to 10 (adherent). The other self-reported questions captured medication management occurrence scores through a 5-point Likert scale rated from never (1) to always (5). Health status was also measured through a 5-point Likert scale question rated from poor (1) to excellent (5). The self-reported adherence questionnaire was used to determine the difference regarding subjective and objective measures of medication adherence between the two groups and overcome the challenge of possible overestimation. A sample of self-reported questionnaire is provided in Additional file 2.

### Data analysis

Descriptive and inferential statistics were used to compare baseline characteristics and adherence among the intervention and control groups. For the baseline, investigators compared the characteristic between the two groups. A Chi-square test of independence was used for categorical variables such as sex, ethnicity, and medication management method. An independent sample t-test was performed for continuous variables such as age, number of chronic conditions, and number of medications. The self-reported medication management and adherence, and health status questionnaire was handled as ordinal data. The between group analysis was conducted using Mann-Whitney U Test. Pairwise, within-group comparison were completed for the self-reported questionnaire at baseline (pre-test) and six month (post-test), using a Wilcoxon Signed Rank Test. Intention-to-treat (ITT) analysis was utilized to keep all of participants’ data in the group they were originally assigned by the randomization process [29]. Statistical tests for inferential statistics was set at 95% confidence level. All the data was entered and analyzed using SPSS Statistics version 25.

## Results

Figure 1 shows the CONSORT flow diagram depicting data from each participant that were included in the study [24]. A total of 50 participants were randomized into the two groups; 24 allocated to intervention and 26 allocated to control. A total of 16 MDS participants and 17 control participants completed the study. The study dropout rate was 33% (*n* = 8) and 35% (*n* = 9) in the intervention and control group, respectively. The number of participants analyzed for ITT was 23 and 25 in the intervention and control group, respectively.

### Sample characteristics

A total of 48 participants completed the baseline (23 intervention and 25 control). Table 1 highlights the baseline comparison of the two groups with statistical analysis for comparison. No statistically significant difference was found between the two groups on most characteristics (*p*-value> 0.05); however, the mean age of the intervention group was higher compared to the control group (63.96 ± 7.86 versus 59.52 ± 5.93, p-value = 0.03).

**Table 1:** Baseline comparison of study participants

Characteristic	Intervention (***n*** = 23)	Control (***n*** = 25)	Statistic	p-value
**AGE** ***(Years)***			−2.22^a^	0.03^d^
Mean (SD)	63.96 (7.86)	59.52 (5.93)		
Age Range	51–82	51–78		
**SEX**			0.01^b^	0.91
Female	16 (69.6%)	17 (68.0%)		
**ETHNICITY**			0.02	0.89
White/Caucasian	21 (91.3%)	24 (96.0%)		
Aboriginal	1 (4.3%)	1(4.0%)		
South East Asian	1 (4.3%)	–		
**LIVING ARRANGEMENT**			0.02	0.88
Alone in residence	8 (34.8%)	12 (48.0%)		
With Spouse	8 (34.8%)	5 (20.0%)		
With family member(s) other than spouse	4 (17.4%)	3 (12.0%)		
Other	3 (13.0%)	5 (20.0%)		
**EDUCATION**			0.03	0.86
Less than High School Diploma	5 (22.7%)	7 (28.0%)		
High School Diploma	4 (18.2%)	6 (24.0%)		
Some College, no degree	11 (50%)	7 (28.0%)		
Bachelor’s Degree	2 (9.1%)	5 (20.0%)		
**EMPLOYMENT STATUS**			0.29	0.59
Employed	1 (4.3%)	1 (4.0%)		
Unemployed	1 (4.3%)	2 (8.0%)		
Retired	11 (47.8%)	6 (24.0%)		
Disabled	10 (43.5)	16 (64.0%)		
**ANNUAL INCOME**			3.61	0.06
< $21,000	9 (39.1%)	16 (66.7%)		
$21,000 - $40,000	9 (39.1%)	6 (25.0%)		
> $40,000	5 (21.7%)	2 (8.3%)		
**CHRONIC HEALTH CONDITIONS**				
Mean (SD)	4.48 (2.19)	4.20 (1.98)	−0.46^a^	0.65
**MEDICATION**				
***Prescriptions***				
Mean (SD)	9.39 (3.73)	10.12 (4.76)	0.59^a^	0.56
***Over-the-counter***				
Mean (SD)	1.68 (1.84)	2.12 (2.74)	0.63^a^	5.53
**HELP TAKING MEDICATION**				
Yes	5 (21.7%)	4 (16.0%)	0.26	0.61
**CAREGIVER**				
Yes	3 (15.8%)	5 (22.7%)	0.31	0.58
**SEEK MEDICATION HELP**^c^			3.09	0.69
Pharmacist	17 (42.5%)	20 (40.0%)		
General Physician	19 (47.5%)	23 (46.0%)		
Specialist	3 (7.5%)	3 (6.0%)		
Homecare Staff (Nurse/HCA)	0	1 (2.0%)		
Other	1 (2.5%)	3 (6.0%)		
**MANAGEMENT METHOD**			1.15	0.28
Blister Pack	5 (26.3%)	12 (52.2%)		
Pill Organizers	9 (47.4%)	4 (17.4%)		
None (pill containers)	2 (10.5%)	6 (26.1%)		
Other	3 (15.8%)	1 (4.3%)		
**MEDICATION TIMES/DAY**			−0.11^a^	0.91
Mean (SD)	2.91 (0.92)	2.87 (1.11)		

^a^Calculated using Independent T-test for Equality of Means

^b^Pearson Chi-square test was used for comparison. For other questions, Chi-Square test for trend was used

^c^Percentages compiled using multiple response analysis and p-value reported using chi-square test

^d^Significant differences at 95% confidence level

**Fig. 2:**
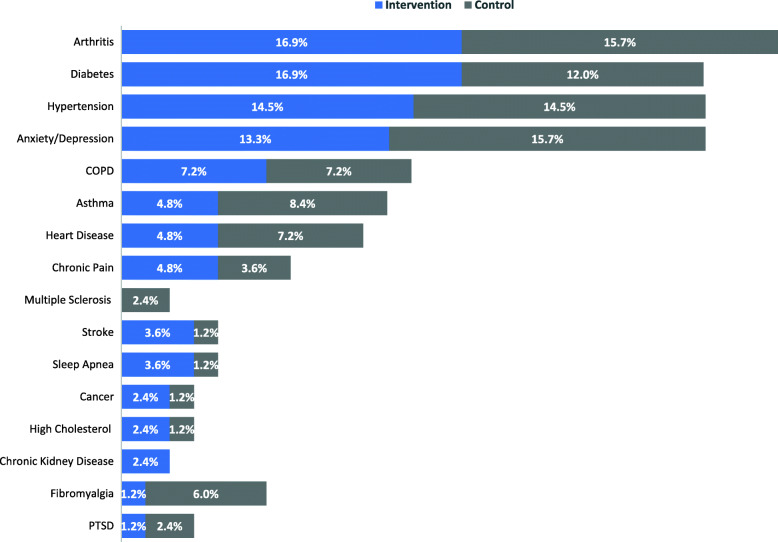
Percentage of common chronic conditions reported by participants (compiled using multiple response analysis)

The most common chronic conditions between the two groups were arthritis, diabetes, hypertension, and anxiety and depression. Figure 2 highlights the percentage of most common chronic conditions reported at baseline between the two groups. Few of the participants indicated needing help with taking medications such as reminders, physical assistance, organizing or sorting medication; the reported percentage was 22% and 16% in the intervention and control group, respectively. They were either assisted by spouse, friend, paid caregiver or homecare staff; some used smartphone applications or phone alarm to help with the reminders. Moreover, 16% in the intervention group and 23% in the control group had a caregiver that provided direct care to them. The caregivers were identified by study participants as either spouse, paid caregiver, or home care staff. The most common medication management method among the intervention group was pill organizers (47%) before starting to use the MDS. Whereas, 52% of the control group were using blister packs for their medication management method. Most of the participants in both groups were on average taking their medications 3 times a day with the most common times of morning, supper and bedtime. Few of the participants (3%) were taking their medications at other times as needed for pain management.

### Self-reported medication management

Table 2 highlights the between-group difference in self-reported scores of medication management and adherence, and health status questionnaire using Mann-Whitney U Test at baseline and six-month. At baseline, the differences in median self-reported medication management scores were comparable between the groups except for two items. First, the distributions differed significantly between the two groups for challenges with taking medications on time rating scores (Mann-Whitney U = 185.5, n_1_ = 23, n_2_ = 25, *p*-value = 0.03). Second, the control group reported lower medication adherence than the intervention group (Mann-Whitney U = 184.5, n_1_ = 23, n_2_ = 25, *p*-value = 0.03).

**Table 2:** Baseline and six-month comparison of self-reported scores

Question	Baseline					6-month				
	*Intervention* (n = 23)	*Control* (n = 25)	*U*	*Z*	*P-value*	*Intervention* (***n*** = 16)	*Control* (***n*** = 15)	*U*	*Z*	*P-value*
In the last 30 days, did you find it challenging to take your medication(s) on time?^a^										
Median (IQR)	3.00 2	3.00 1	185.5	−2.21	0.03^d^	1.00 2	2.00 2	99.5	−0.87	0.38
In the last 30 days, did you miss taking any medication(s)?^a^										
Median (IQR)	2.00 2	3.00 2	207.5	−1.74	0.08	1.00 1	2.00 1	64.0	−2.49	0.01^d^
In the last 30 days, please rate your medication adherence (taking your medication on time) on a scale of 1 to 10.^b^										
Median (IQR)	8.00 2	7.00 4	184.5	−2.16	0.03^d^	9.00 2	9.00 3	100.0	−0.83	0.41
In general, how would you say your health is in the last 30 days?^c^										
Median (IQR)	2.00 1	2.00 1	218.5	−1.58	0.12	3.00 1	2.00 2	86.0	−1.41	0.16

*IQR* Interquartile Range

^a^Likert scale was used to obtain occurrence scores (1 = Never; 2 = Rarely; 3 = Sometimes; 4 = Often; 5 = Always)

^b^Likert scale was used to obtain adherence scores (1 = Least Adherent; 10 = Most Adherent)

^c^Likert scale was used to obtain health status quality scores (1 = Poor; 2 = Fair; 3 = Good; 4 = Very Good; 5 = Excellent)

^d^Significant differences at 95% confidence level

**Fig. 3:**
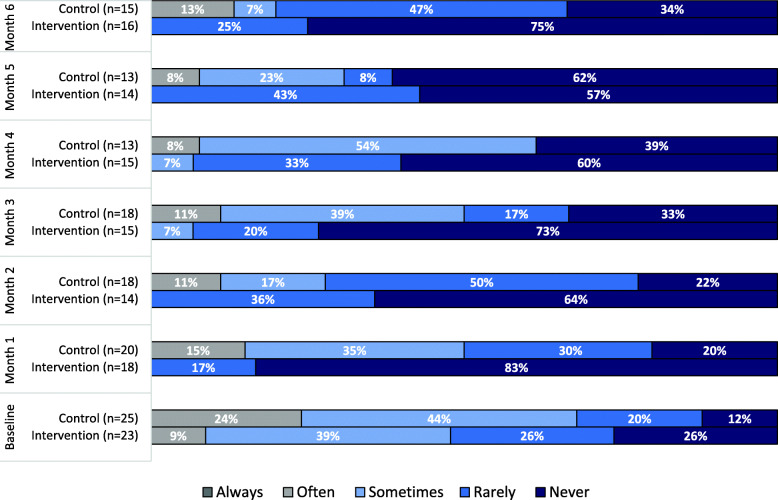
Percentage of participants reporting missing their medications during the study

From baseline to six-months, both groups reported less challenges with taking their medications on time. Figure 3 shows the percentage of participants reporting missing their medications in both groups at baseline and follow-ups. At baseline, participants that reported missing their medication (as sometime or rarely) was 65% and 64% in intervention and control group, respectively. However, as the intervention participants started using the MDS, the percentage of never missing their medication increased from 26% at baseline compared to 83% at one-month. The desired behavior was sustained during the study with the help of the MDS as only 25% of participants reported rarely missing their medications at six-month compared to 47% in the control group. Also, 13% of the control participants reported often missing their medication compared to 0% in the intervention group at six-month. At six-month comparison (Table 2), the intervention group was missing their medications a lot less compared to the control group (Mann-Whitney U = 64.0, n_1_ = 16, n_2_ = 15, *p*-value = 0.01).

Table 3 compiles the number and percentages of the six self-reported reasons for participants missing their medications at baseline and during the follow-ups between the two groups using multiple response analysis. The two major reasons for participants missing their medications were related to busyness and forgetfulness. At the time of baseline completion, the number of participants reporting the two reasons for missing their medications was very similar between the two groups; in fact, the reported percentages were slightly higher in the intervention group. During the six months of the study, the number of MDS users reporting forgetfulness as a reason for missing their medication reduced to half (n = 8) compared to baseline (n = 16). Meanwhile, in the control group, the number of reported reasons for forgetfulness and busyness was higher during follow-ups compared to baseline.

**Table 3:** Self-reported reason(s) for missing medication(s)

Reason	Baseline		Follow-ups (Combined Responses)^a^	
	*Intervention* (***N*** = 35)	*Control* (***N*** = 45)	*Intervention* (***N*** = 19)	*Control* (***N*** = 74)
Forgot to take the medication(s)				
n (%)	16 (45.7%)	17 (37.8%)	8 (42.1%)	42 (56.8%)
Were too busy to take the medication(s)				
n (%)	12 (34.3%)	13 (28.9%)	11 (57.9%)	23 (31.1%)
Did not want to take the medication(s)				
n (%)	2 (5.7%)	5 (11.1%)	0 (0.0%)	5 (6.8%)
Had too many medication(s) to take				
n (%)	2 (5.7%)	4 (8.9%)	0 (0.0%)	1 (1.4%)
Had to take medication(s) at too many times in a day				
n (%)	3 (8.6%)	5 (11.1%)	0 (0.0%)	3 (4.1%)
Did not have enough information on or feel unsure of taking the medication(s)				
n (%)	0 (0.0%)	1 (2.2%)	0 (0.0%)	0 (0.0%)

Reason(s) for missing medication question was only asked if participants selected response other than “never” for missed medication(s)

Numbers in the table are reported using Multiple Response Analysis as participants could select more than one option (check all that apply). ‘N’ represents total responses and not total number of completed questionnaires’

^a^Follow-ups include combined responses from all six months of data collection due to low responses within each month

Table 4 analyzes the within-group difference in median rating scores of self-reported medication management and adherence, and health status questionnaire completed at baseline (pre-test) and six-month (post-test) using a Wilcoxon Matched-Pairs Signed Rank Test. Both groups found it less challenging to take their medications on time from baseline to six-month as the post-test rating scores were statistically significantly lower than the pre-test scores. Also, participants were asked to rate if they took their medications at a different time than prescribed. The control group showed a significant decrease in rating score from baseline to six-month (Z = -2.43, M_1_ = 3, M_2_ = 1, p-value< 0.01). Moreover, when the participants were asked about missing their medications, both groups showed statistically significant findings. MDS participants were missing their medications less frequently from baseline to six-month with median responses of rarely to never, respectively (Z = -2.70, M_1_ = 2 M_2_ = 1, *p*-value< 0.01). Whereas, the control group shifted from sometimes to rarely for missing their medications from baseline to six-month (Z = -2.23, M_1_ = 3 M_2_ = 2, p-value = 0.03).

**Table 4:** Wilcoxon Matched-Pairs Signed Rank Test comparing pretest and posttest self-reported scores

Question	n	Baseline – M_**1**_ Median (IQR)	6-month - M_**2**_ Median (IQR)	Z	p-value
In the last 30 days, did you find it challenging to take your medication(s) on time?^a^					
*Intervention*	16	3.00 (2)	1.00 (2)	−2.28	0.02^d^
*Control*	15	3.00 (1)	2.00 (2)	−2.78	< 0.01^d^
In the last 30 days, did you take your medication(s) at a different time than prescribed?^a^					
*Intervention*	16	3.00 (2)	2.00 (2)	−1.35	0.18
*Control*	15	3.00 (3)	1.00 (1)	−2.43	< 0.01^d^
In the last 30 days, did you miss taking any medication(s)?^a^					
*Intervention*	16	2.00 (2)	1.00 (1)	−2.70	< 0.01^d^
*Control*	15	3.00 (1)	2.00 (1)	−2.23	0.03^d^
In the last 30 days, please rate your medication adherence (taking your medication on time) on a scale of 1 to 10.^b^					
*Intervention*	16	8.00 (2)^e^	9 (2)	−2.65	< 0.01^d^
*Control*	15	7.00 (4)^e^	9.00 (3)	−1.79	0.07
In general, how would you say your health is in the last 30 days?^c^					
*Intervention*	16	2.50 (1)	3.00 (1)	−2.13	0.03^d^
*Control*	15	2.00 (0)	2.00 (2)	−0.83	0.41

*IQR* Interquartile Range

^a^Likert scale was used to obtain occurrence scores (1 = Never; 2 = Rarely; 3 = Sometimes; 4 = Often; 5 = Always)

^b^Likert scale was used to obtain adherence scores (1 = Least Adherent; 10 = Most Adherent)

^c^Likert scale was used to obtain health status quality scores (1 = Poor; 2 = Fair; 3 = Good; 4 = Very Good; 5 = Excellent)

^d^Significant differences at 95% confidence level

^e^The reported values at baseline are different than Table 2, Table 5 and Fig. 5 because the Wilcoxon Matched-Pairs Signed Rank Test only included recoded values at pre-post measurement and missing values are excluded

### Adherence data

**Fig. 4:**
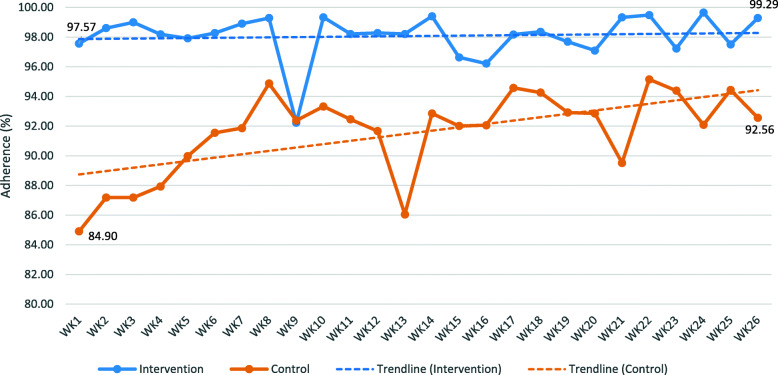
Recorded weekly medication adherence with trendline

For the recorded adherence, the control group was significantly different than the intervention group. The average recorded adherence over 26 weeks was significantly higher in the intervention group than the control group (98.35 ± 2.15% versus 91.17 ± 9.76%, p < 0.01). Figure 4 depicts the weekly pattern of recoded medication adherence between the two groups. The intervention group had an average adherence of 97.57% ± 5.03% in the first week of using the MDS and 99.29% ± 2.00 in the last week. The control group started at 84.90% ± 22.02 in the first week and gradually increased to 92.56% ± 14.48% in the last week. It appears that in the first 14 weeks, the intervention and control groups were significantly different for all except for two weeks (week 9 and week 11), *p* < .05. The decrease in medication adherence for the intervention group in week 9 is explained by few participants not taking their medications within the two hour window and few others withdrawing from the study with some non-adherent values for the last day. Comparatively, the control group observed a sudden decrease in medication adherence in week 13 due to few participants reporting higher occurrence of missed dose than other weeks. For weeks 15 through 26, the difference between the two groups was not significant. While, medication adherence in the intervention group had remained steady (ranging between 97 and 99%). The weekly comparison of adherence showed that the difference between the two groups was minimized after week 15. Regarding the self-reported medication adherence, when comparing the pre-post analysis (Table 4), the intervention group showed a significant increase in average medication adherence from baseline to six-month (Z = -2.65, *p* < 0.01). The control group showed a non-significant increase (Z = -1.79, p = 0.07).

**Fig. 5:**
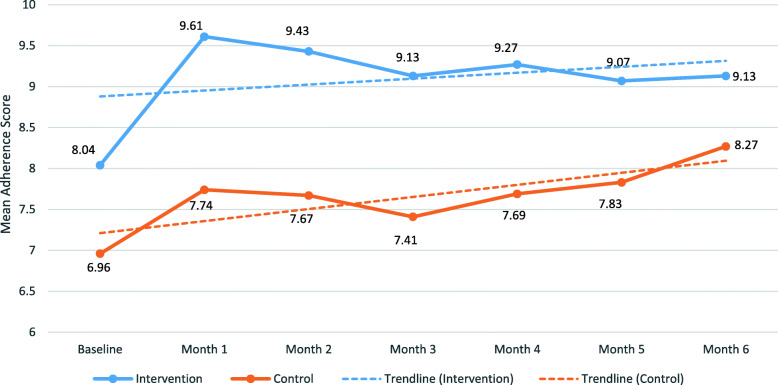
Self-reported medication adherence with trendline

Figure 5 depicts the pattern of self-reported mean medication adherence scores between the two groups showing higher adherence scores in the intervention group with an upward linear trend. Table 5 shows scores were higher in the intervention group compared to control for all six months. Comparing Figs. 4 and 5, there is no evidence of overestimation for self-reported medication adherence scores. In fact, the monthly self-reported scores are lower than the actual recorded adherence percentage between the two groups. Table 5 also highlights the self-reported rating for participants’ medication management method effectiveness (reported on a 10-point scale). The effectiveness scores were only collected during follow-ups after intervention participants started using the MDS and not at baseline. During the six months of the study, the effectiveness scores were higher in the intervention group compared to the control group.

**Table 5:** Self-reported rating scores of effectiveness and medication adherence during the study

Question	Baseline	Month 1	Month 2	Month 3	Month 4	Month 5	Month 6
	Mean (SD)	Mean (SD)	Mean (SD)	Mean (SD)	Mean (SD)	Mean (SD)	Mean (SD)
On a scale of 1–10, how would you rate MDS effectiveness in helping you manage your medications in the last 30 days?^a^							
*Intervention*	N/A^c^	*n* = 18 8.61 (2.23)	*n* = 14 8.93 (2.17)	n = 15 8.60 (2.23)	n = 15 8.67 (2.44)	n = 14 9.64 (0.50)	n = 16 9.25 (1.34)
On a scale of 1–10, how would you rate your current medication management method’s effectiveness in helping you manage your medications in the last 30 days? ^a^							
*Control*	N/A^c^	n = 18 7.39 (2.17)	*n* = 17 7.59 (2.18)	n = 16 8.19 (2.04)	*n* = 13 6.92 (3.04)	n = 13 7.85 (2.58)	n = 15 7.80 (2.68)
In the last 30 days, please rate your medication adherence (taking your medication on time) on a scale of 1 to 10.^b^							
*Intervention*	n = 23 8.04 (1.61)	n = 18 9.61 (0.70)	n = 14 9.43 (0.76)	n = 15 9.13 (1.19)	n = 15 9.27 (1.10)	n = 14 9.07 (1.14)	n = 16 9.13 (0.81)
*Control*	n = 25 6.96 (1.72)	n = 19 7.74 (1.66)	n = 18 7.67 (1.78)	n = 17 7.41 (2.50)	n = 13 7.69 (2.02)	*n* = 12 7.83 (1.90)	n = 15 8.27 (2.09)

^a^Likert scale was used to obtain effectiveness rating scores (1 = Extremely Ineffective; 10 = Extremely Effective)

^b^Likert scale was used to obtain adherence scores (1 = Least Adherent; 10 = Most Adherent)

^c^Effectiveness rating scores weren’t collected at baseline

### Self-reported health status

**Fig. 6:**
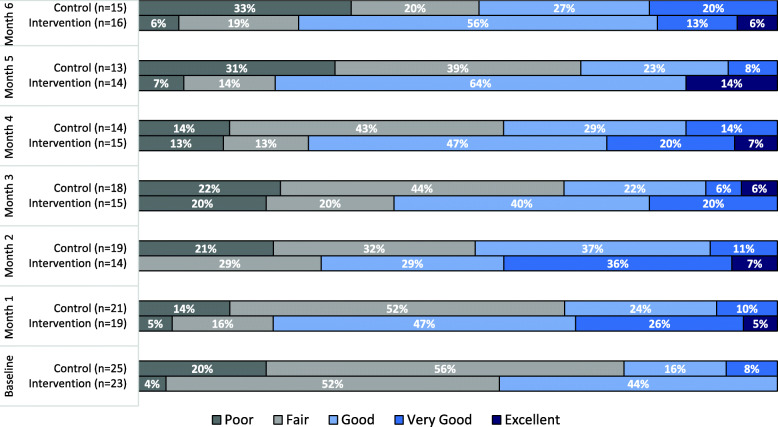
Self-reported health status by participants during the study

Figure 6 highlights the percentage of self-reported health status during the study for intervention and control participants. At baseline, the majority of the participants reported their health as fair; 52% in the intervention group and 56% in the control group. None of the participants from the two groups reported their health as excellent. Furthermore, only 8% of the control group participants reported their health as very good, while no participants in the intervention group reported that. The percentage of control participants reporting their health as poor increased from 14% at one-month to 33% at six-month. Whereas, the percentage of control participants reporting their health as fair decreased from 52% at one-month to 20% at six-month. In the intervention group, the percentage of participants reporting their health as very good (0 to 13%) or excellent (0 to 6%) increased from baseline to six-month. When comparing the pre-post analysis regarding health status (Table 4), the intervention group had a statistically significant increase in their perceived health status from baseline to six-month (Z = -2.13, p-value = 0.03). Meanwhile, no observable statistical difference in health improvement was evident for the control group from baseline to six-month (Z = -0.83, *p*-value = 0.41).

## Discussion

Medication nonadherence in the older population due to polypharmacy [30] and complex medication regimens is a major public health issue. Investigators conducted a pilot RCT to examine whether an in-home medication dispensing technology can improve medication adherence of users. The results of the study demonstrate that the MDS can support medication management in community-dwelling older adults living with chronic conditions. The findings validated existing qualitative evidence in which older adults claimed the use of medication dispensing technology improved their medication adherence [15, 16, 19].

The average medication adherence recorded by the MDS over the six-month study duration was 98.35% in this study. Other studies related to medication dispensing technology reported participants’ average adherence from 97 to 98% [31, 32]. The evidence suggests that medication dispensing technology can promote extremely high adherence in patients that are non-compliant with their medication regimen. The trend of recorded medication adherence further revealed that the MDS provided users immediate improvement and consistent adherence over time. A similar pattern was observed by intervention participants’ self-rating of medication adherence at baseline and follow-ups. Evidence of improvements was corroborated by participants’ self-rated increase in adherence, and decrease in missed medications and difficulties in taking medications on time by the end of the study. The findings from this study, in addition to evidence of existing studies, show that medication dispensing technology can improve medication adherence in older adults within the first week or month, and the high adherence is likely to be maintained over the six month period [17, 31, 33, 34]. Two studies have suggested the possibility of an even longer maintenance of medication adherence for community-dwelling older adults, at two to over three years of use [18, 34].

In comparison, the control group had a consistently lower and wider range of medication adherence. Interesting, there was a gradual increase in self-recording and self-rating as the study progressed. One explanation could be that the task of daily medication tracking had inadvertently made the control participants more aware of their medication regimens and improved their adherence over time. Also, because the control group had a significantly lower self-rated medication adherence to begin with, due to chance [35], they had more room for improvement. The gradual increase in medication adherence in the control group may explain the lack of significant differences in recorded adherence between intervention and control patients after week 14, and the lower self-reported challenges in taking medication at 6-month, compared to baseline (Table 4). Another intriguing finding is that the control group’s highest data point of self-rated medication adherence at six-month (8.27 out of 10 which can be converted to 82.7%; Table 5) was lower than the lowest data point of self-recorded medication adherence at week 1 (84.9%; Fig. 4). Since the study was open-label, this indicates that the control group believed they were not as adherent as they were. It may be that non-technological intervention or the lack of intervention gave them less confidence in their ability to take medications according to their regimens. These findings highlight the fact that the participants weren’t overestimating their self-reported adherence due to Hawthorne effect of being observed.

Overall, this study showed that the MDS induced better adherence in intervention participants than non-technological medication management methods used by control participants. This is congruent with existing research that concluded the superiority of medication dispensing technology in decreasing missed doses for older adults over self-management of medications [31], and the use of pill organizers [31, 32]. One study conducted in the Netherlands also reported that older adults already using the technology had higher adherence than non-users [36].

Study participants who reported medication adherence issues at baseline and follow-ups mainly attributed it to forgetfulness and busyness. Behavioural strategies have been recommended to intervene with these commonly reported, yet preventable barriers in individuals with long-term pharmacotherapy commitments [37]. The higher medication adherence in MDS users compared to non-users could be mainly attributed to the audio and visual reminders [17, 18, 33, 34]. The alerts emitted from the MDS served as behavioural cues to induce remembering and prevent distraction. Other features of the MDS may have also contributed to improved outcomes. Participants’ awareness of pharmacy adherence and symptom monitoring through the MDS may have increased their effort to be more adherent. Pre-organized mediation packaging and early dispensing made managing polypharmacy easier for participants. The early dispensing feature provided greater flexibility and independence for daily routine to MDS users. However, the *p*-value comparing the median rating score from baseline to six-month for MDS participants taking their medications at a different time than prescribed was not statistically significant (Z=-1.35, p-value = 0.18). One possible explanation for this finding is when using the early dispensing feature, few of the participants thought they took their medications at a different time than prescribed as opposed to the reminder time. Nevertheless, effective interventions tend to integrate multiple components and features [38, 39]. The combination of features provided by the MDS (including the early dispensing option) may have produced stronger, additive effects compared to the simple, non-technological interventions used by the control participants for self-management of medication.

Current evidence of health outcomes associated with the use of medication dispensing technology has been scarce and mixed. Some studies suggest it lowers the number of hospitalization, emergency department visits and physician appointments, and improves self-reported physical and mental health [15, 31, 32]. Conversely, a study found that under a nurse care coordination program, the use of a medication dispensing device did not improve depression, cognition, quality of life, and functional status, whereas patients using a pill organizer did [40]. In this study, the investigators found the MDS improved the percieved health status of users over time, whereas the control partcipants did not perceive improvements in health. Nevertheless,  the study was unable to measure the impact of medication dispensing technology on the actual health outcomes of older adults, which was beyond the scope of this study.

### Limitations

This study had a few limitations. The study was open-label where knowledge about group assigned to participants after randomization was not withheld from the participants or the research team. The lack of blinding could have resulted in potential bias leading to higher self-reported adherence and perceived health in the intervention group. Also, although the outcome was the same for both groups (i.e., medication adherence), different methods were used to collect adherence data in the intervention and control groups. The self-tracking method used in the control group might not be fully accurate as a result of recall bias and completing calendars at different times or days. However, the research team tried to reduce bias by sending personalized calendars to control participants and used the same approach as MDS users to calculate adherence. Self-reported adherence scores were also used to supplement the recorded adherence.

Investigators encountered some data collection issues during the study. The research team was unable to obtain complete data from some participants. Intermittent data were collected because some participants were unavailable for all monthly follow-up phone calls; control participants were not consistently sending in medication tracking calendars; and, a few intervention participants experienced technical issues with their MDS, causing short periods of missed recordings through the electronic monitoring system. Investigators also experienced a high dropout rate with roughly equal number of dropouts in both control and intervention arms. This may be due to the content of the intervention and the design of the study, such as the long data collection period and frequency of data collection (i.e., monthly follow-up phone calls and daily calendar tracking). The investigators followed the guidelines of the ITT approach to minimize bias due to missing responses [41]. Lastly, the sample size in this study was small and caution needs to be taken in the interpretation of the results [42, 43].

### Future research direction

In this study, the measured impact of mediation dispensing technology on health status was strictly perceptual and may not correspond to objective measures of clinical health outcomes. Nevertheless, the MDS Bluetooth function could be used to capture daily vital sign data and health outcomes. In theory, the use of medication dispensing technology and subsequent improvement in medication adherence have the potential to prevent or alleviate worsening health outcomes for older adults with chronic conditions and adherence issues. The use of medication dispensing technology, thus, may have significant implications for the healthcare system, given non-adherent older adults attribute to 11% of hospital admissions [44]. Future research should examine whether using medication dispensing technology attributes to changes in individuals’ health outcomes and healthcare system utilization, and whether improved medication adherence mediates the changes. Investigators plan to report on interviews conducted with MDS users and healthcare providers at the end of the study to obtain further insights about the topic.

Medication dispensing technology has the potential to decrease healthcare system utilization, allow healthcare resources to be used more effectively, and, ultimately, decrease healthcare costs. Future, large-scale studies are warranted to ascertain medication dispensing technology’s impact on different groups of older population and the healthcare system.

## Conclusion

The evidence from this study suggests that the MDS can be an effective, long-term solution to medication non-adherence in community-dwelling older adults experiencing chronic conditions and taking multiple medications. The technology induces better consistency and improvement in medication taking behaviour than simple, non-technological intervention. Future, large-scale research is warranted to demonstrate medication dispensing technology’s potential in enhancing older patients’ health outcomes, and reducing healthcare system utilization and costs.

## Supplementary Information

**Additional file 1.**

**Additional file 2.**

## Data Availability

All data collection, management and storing procedures complied with the *Health Information Act and the Freedom of Information and Privacy Act*. The datasets generated and analyzed during the current study are not publicly available due to ethics and organizational data sharing restrictions. Baseline assessment and monthly follow-up questionnaires can be shared with interested parties upon request.

## Abbreviations

MDS: Medication Dispensing System
RCT: Randomized Controlled Trial
ITT: Intention-to-Treat
CONSORT: Consolidated Standards of Reporting Trials
REDCap: Research Electronic Data Capture
