# Supplementary material for: Medication adherence support of an in-home electronic medication dispensing system for individuals living with chronic conditions: a pilot randomized controlled trial
Source: BMC Geriatr. 2021 Jan 14;21:56. doi: 10.1186/s12877-020-01979-w (PMC7807760; doi:10.1186/s12877-020-01979-w)
Supplement: Supplementary file 1 — Additional file 1. [file 12877_2020_1979_MOESM1_ESM.docx]

# Additional file 1

Daily Adherence = X 100%

Example: Assume user has 4 dispenses per day including morning, noon, supper and bedtime but the user does not take their bedtime doses.

| **Dispensing Time(s)** | **Dispensing Adherence Value** |
| --- | --- |
| Morning | 100 |
| Noon | 100 |
| Supper | 100 |
| Bedtime | 0 |
| ***Daily Adherence*** | ***300/4 x 100% = 75%*** |
