# Supplementary material for: Medication adherence support of an in-home electronic medication dispensing system for individuals living with chronic conditions: a pilot randomized controlled trial
Source: BMC Geriatr. 2021 Jan 14;21:56. doi: 10.1186/s12877-020-01979-w (PMC7807760; doi:10.1186/s12877-020-01979-w)
Supplement: Supplementary file 2 — Additional file 2. [file 12877_2020_1979_MOESM2_ESM.docx]

**Additional file 2**

We know taking medications on time can be difficult to remember at times. Therefore, the following questions are designed to help us understand how you are managing your medication(s). Please think back on **the last 30 days** while you consider the following situations:

|  | **Always** | **Often** | **Sometimes** | **Rarely** | **Never** |
| --- | --- | --- | --- | --- | --- |
| 1. In the last 30 days, did you refill your medication(s) on time, as scheduled? | O | O | O | O | O |
| 1. In the last 30 days, did you find it challenging to take your medication(s) on time? | O | O | O | O | O |
| 1. In the last 30 days, did you miss taking medication(s)? | O | O | O | O | O |
| 1. In the last 30 days, did you take your medication(s) at a different time than prescribed? | O | O | O | O | O |
| 1. In the last 30 days, did you notice that you had taken the same medication(s) twice? | O | O | O | O | O |
| 1. In the last 30 days, did you rely on someone or technology (e.g alarm) to remind you to take medication(s)? | O | O | O | O | O |

1. In the last 30 days, please tell the reason(s) for missing your medication(s)? (check all that apply)

☐ Forgot to take the medication(s)

☐ Were too busy to take the medication(s)

☐ Did not want to take the medication(s)

☐ Had too many medication(s) to take

☐ Had to take medication(s) at too many times in a day

☐ Did not have enough information on or feel unsure of taking the medication(s)
☐ Other reason(s) (please specify): _----------------------------------------------------------------------------------------------------------------------------_

1. In the last 30 days, please rate your medication adherence (refers to taking your medication on time) on a scale of 1 to 10. Please circle the appropriate number, where 1 indicates being least adherent and 10 indicates being most adherent.

1 2 3 4 5 6 7 8 9 10

1. In general, how would you say your health is?

Poor

Fair

Good

Very Good

Excellent
